# First-in-human PET imaging and evaluation of melanin-targeted [^18^F]DMPY2 in malignant melanoma patients

**DOI:** 10.7150/thno.109243

**Published:** 2025-05-07

**Authors:** Yi Yang, Ming Zhou, Shuang Zhao, Tingting Long, Na Chen, Xingming Wang, Yulai Li, Xiaozhen Chen, Junchen Chen, Xinqiong Huang, Dengming Chen, Juan Su, Shuo Hu, Xiang Chen

**Affiliations:** 1 Department of Nuclear Medicine, Xiangya Hospital, Central South University, Changsha, Hunan, China.; 2 Department of Dermatology, Xiangya Hospital, Central South University, Changsha, Hunan, China.; 3 Department of Oncology, Xiangya Hospital, Central South University, Changsha, Hunan, China.; 4 Furong Laboratory, Changsha, Hunan, China.; 5 National Clinical Research Center for Geriatric Disorders, Xiangya Hospital, Changsha,Hunan, 410008, China.; 6 Hunan Key Laboratory of Skin Cancer and Psoriasis, Changsha, Hunan, China.; 7 National Engineering Research Center of Personalized Diagnostic and Therapeutic Technology, Changsha, Hunan, China.; 8 Key Laboratory of Biological Nanotechnology of National Health Commission, Xiangya Hospital, Central South University, Changsha, Hunan, China.

**Keywords:** [^18^F]DMPY2, malignant melanoma (MM), melanin, PET imaging, early detection

## Abstract

**Aim:** Early diagnosis and accurate malignant melanoma (MM) staging are significant and decisive in clinical practice. [^18^F]DMPY2 is a promising PET tracer *in vivo* with high affinity and selectivity for melanin. The study aims to investigate the biodistribution and radiation dosimetry in healthy volunteers, and the potential clinical application of [^18^F]DMPY2 in MM patients.

**Materials and Methods**: [^18^F]DMPY2 was synthesized via a one-pot reaction. The biodistribution, radiation dosimetry, and probe safety were estimated in three healthy volunteers. Thirty-one MM patients underwent [^18^F]DMPY2 and/or [^18^F]FDG PET/CT scans to explore the clinical use in early detection of melanoma metastasis. Diagnostic performance was assessed in fifty-one LN basins of twenty-seven MM patients after surgery, comparing PET uptake with pathological results.

**Results**: [^18^F]DMPY2 was well tolerated by healthy volunteers and MM patients. The calculated effective dose of [^18^F]DMPY2 was 0.0122 mSv/MBq. In MM patients, we observed prominent [^18^F]DMPY2 tumor uptake and high tumor-to-background ratios in primary tumors. [^18^F]DMPY2 showed superior diagnostic performance in lymph node metastases compared to [^18^F]FDG, with sensitivity, specificity, accuracy, positive and negative predictive values of 66.7%, 100%, 88.9%, 100% and 85.7%, respectively, versus 50%, 42.9%, 46.7%, 50% and 42.9% for [^18^F]FDG. Additionally, [^18^F]DMPY2 PET imaging had a unique advantage in distinguishing [^18^F]FDG false-positive lesions.

**Conclusion**: [^18^F]DMPY2 is a safe and well-tolerated melanin PET tracer and can be a powerful imaging tool for early detection and clinical staging of patients with MM.

## Introduction

Malignant melanoma (MM) is one of the most lethal cutaneous tumors characterized by high malignancy, early metastasis, increasing incidence rate and poor prognosis. In the United States, there are 100,640 estimated new cases of cutaneous melanoma diagnosed in 2024, ranking fifth in incidence among both male and female estimated new cases 1. Advanced melanoma patients usually have poor prognosis. However, early detection and precise surgical excision of melanoma can boost the 5-year overall survival rate up to 98%. Cutaneous melanoma has a high risk of an estimated 30% or 50% of regional lymph node(LN) an estimated 30% or 50% of regional lymph node (LN) metastasis occurring with depth of invasion of over 2 mm or 4 mm, respectively 2. While acral melanoma represents only 1-9% of cases in Caucasians, it represents the majority (58%) of melanomas in Asian populations, with a higher likelihood of LN metastasis 3. In melanoma patients, LN metastasis requires a shift from sentinel LN biopsy to comprehensive LN dissection and upgrades the disease to Stage III, necessitating adjuvant therapies like targeted or immunotherapy. Early and accurate evaluation of LN metastasis is critical for optimizing surgical strategies and guiding systemic therapy selection, ensuring effective postoperative surveillance, and ultimately improving patient outcomes and management. This underscores the importance of effective diagnostic tools for early detection, accurate staging, and management of melanoma.

Current imaging methods, such as CT, MRI, and ultrasound are inadequate for accurately assessing LN metastases 4. PET imaging, a non-invasive technique, has shown clinical value in diagnosing and managing various cancers 5. [^18^F]FDG PET/CT is used for initial staging, restaging, evaluation of tumor response to therapy and follow-up surveillance in stage Ⅲ or Ⅳ melanoma 6, 7. However, for patients with melanoma *in situ* or stage I or II, [^18^F]FDG PET/CT has limitations in specificity and sensitivity 8. Furthermore, it is challenging to visualize hidden metastatic lesions, particularly those with diameters smaller than 1 cm in the liver, and brain 9. Occasionally, it might be difficult to differentiate between inflammatory lesions and tumor foci using [^18^F]FDG PET/CT, often leading to false-positive results. Therefore, there is an urgent clinical need to discover a new PET tracer targeting melanoma to enhance the visualization and characterization of melanoma lesions.

Several specific PET imaging probes have been developed for melanoma diagnosis, but their clinical applications are limited due to drawbacks such as low tumor affinity, high background distribution, and aggregation in the liver 10, 11. Melanin presents a promising imaging target as it is found in over 90% of melanomas and is closely related with tumor heterogeneity and therapy response. As early as 2009, PET probes targeting melanoma, such as [^18^F]DAFBA and [^18^F]FBZA, were designed and demonstrated good diagnostic efficacy in preclinical PET imaging 12, 13. Although it has not been validated at the clinical level, [^18^F]MEL050 showed superior uptake in both primary and lung metastatic lesions in mouse melanoma models compared to [^18^F]FDG 14. Clinical studies of melanin-targeted probes, including [^18^F]P3BZA PET/CT and [^18^F]PFPN PET/MR, have been conducted in melanoma patients 15, 16. Both probes could visualize primary melanoma lesions, with [^18^F]P3BZA PET/CT showing significantly higher SUVmax uptake than [^18^F]FDG PET/CT. However, [^18^F]PFPN PET/MR demonstrated superior performance in detecting distant metastases compared to [^18^F]FDG PET/CT. N-(2-(dimethylamino) ethyl)-5-[^18^F]fluoropicolinamide ([^18^F]DMPY2), a radiolabeled and optimized benzamide analog targeting melanin, has shown high affinity and selectivity for melanoma, rapid clearance, and reduced background noise in B16F10 primary tumor, lung, and LN metastasis models 17.

Given the superior performance of [^18^F]DMPY2 in detecting metastatic melanoma and its potential for clinical application, we designed this first-in-human clinical study with two major purposes. First, we aimed to investigate the biological distribution, radiation dosimetry, and safety of [^18^F]DMPY2 in healthy volunteers. Second, we explored its clinical application in the early diagnosis of primary melanoma and LN metastasis.

## Materials and methods

### Chemistry and radiolabeling

The precursor 5-Bromo-N-(2-(dimethylamino)ethyl)picolinamide of [^18^F]DMPY2 was acquired from Biochempartner (Shanghai, China) with a high purity (> 95%) and other commercial reagents and solvents were used without further purification. [^18^F]FDG was supplied by the Department of Nuclear Medicine, Xiangya Hospital, Central South University and [^18^F]DMPY2 was synthesized as described previously 17. [^18^F]DMPY2 was obtained in 23 ± 3.7% (decay-corrected; n = 8) radiochemical yield, with a radiochemical purity of > 99%, and molar activity of 23 ± 2.5 GBq/μmol (n = 5) starting from 37 GBq of [^18^F]fluoride. Radio-HPLC purification and trace of [^18^F]DMPY2 are shown in Figure S1. Detailed synthesis and analysis methods are described in Supporting Information.

### Study population

The prospective clinical study was approved by the Ethics Committee of Xiangya Hospital (No. 202104001) and performed in accordance with the 1964 Helsinki Declaration and its later amendments or comparable ethical standards. All participants signed informed consent forms. Three healthy volunteers (age range, 36-41 years; two men and one woman; Table 1) were enrolled in safety, dose measurement, and biological distribution studies. Safety data were collected before and after scanning, vital signs (blood pressure, pulse rate, respiratory rate, and temperature) were monitored, physical examinations, electrocardiograms, and routine blood tests, were performed, and liver and renal functions were assessed. Any abnormal or adverse clinical symptoms were also recorded. Additionally, thirty-one operable patients with pathologically confirmed malignant melanoma (age range 17-87 years; fifteen men and sixteen women; average weight 57.74 ± 5.86 kg) were recruited to assess the preliminary clinical application of [^18^F]DMPY2.

### PET/CT acquisition protocol and image interpretation

[^18^F]FDG and [^18^F]DMPY2 doses were administered intravenously based on patient weight (3.7 MBq [0.1 mCi]/kg for FDG; 3.6-3.8 MBq [0.09-0.11 mCi]/kg for DMPY2). Whole-body (top of the skull to toe) low-dose CT scans (120 kV; automatic mAs; pitch, 1:1; slice thickness, 3.75 mm; matrix, 512 × 512) were performed. MM patients were required to fast for at least 6 h and maintain normal blood glucose levels for [^18^F]FDG PET/CT evaluation. For the three volunteers, whole-body PET scans (15.7-cm axial and 70-cm transaxial fields of view) were conducted at 10, 30, 60, 90 and 120 min after tracer injection with a 2-minute acquisition duration on a General Electric Discovery PET/CT 690 Elite scanner (General Electric Healthcare, Waukesha, Wis). Seventeen MM patients underwent paired [^18^F]DMPY2 PET/CT and [^18^F]FDG-PET/CT scans after IV administration, with each bed position lasting two min. [^18^F]FDG PET/CT scans were obtained within one week of [^18^F]DMPY2 PET/CT. An additional fourteen patients underwent only [^18^F]DMPY2 PET/CT.

The PET visual interpretation and SUV (SUVmean or SUVmax) values were independently evaluated by two experienced nuclear medicine physicians using an Advantage Workstation (AW 4.6; General Electric Healthcare). Discrepancies were resolved by consensus. PET uptakes of primary tumors, drainage LN, other metastases, and major organs were calculated by drawing volume for regions of interest (ROIs). To enhance the comparability of SUVmax values, standardized SUVmax values were calculated by dividing the raw SUVmax by the SUVmean of the aortic arch. The diagnostic performance of [^18^F]DMPY2 PET/CT and [^18^F]FDG-PET/CT imaging was estimated by comparing PET image results with pathological findings from surgery or biopsy, which is regarded as the gold standard.

### Biodistribution and dosimetry

The SUVmean of each major organ was used to calculate the biodistribution of [^18^F]DMPY2 in healthy volunteers. Three-dimensional ROIs in PET/CT images were drawn in Hybrid-Dosimetry software (Hermes Medical Solutions, 2020, Sweden). The estimated absorbed radiation dosimetry for different organs was calculated using the OLINDA/EXM software (version 2.2, Hermes Medical Solutions, 2020, Sweden). ROIs included brain, liver, lung, heart, spleen, kidney, stomach, pancreas, gallbladder, intestine, red marrow, thyroid, blood, muscle. In females, breasts, ovaries, and uterus were also measured, while prostate and testes were analyzed in males.

### Statistical analysis

SUVmax values between the two groups were compared with Prism software (GraphPad) using the two-tailed unpaired Student's t-test. ROC curves were conducted using MedCalc statistical software. Continuous variables are presented as mean only or mean ± standard error of the mean (s.e.m.). Statistical significance was set at p < 0.05. Symbols *, **, ***,**** represented p values < 0.05, < 0.01, < 0.001 and < 0.0001, respectively.

## Results

### Safety, biodistribution and dosimetry of [^18^F]DMPY2

No adverse events or clinically detectable pharmacological effects were observed in any patients during the clinical study. No significant changes were found in vital signs, laboratory test results, or electrocardiograms, demonstrating the safety and tolerability of the [^18^F]DMPY2 tracer in healthy volunteers.

**Figure 1:**
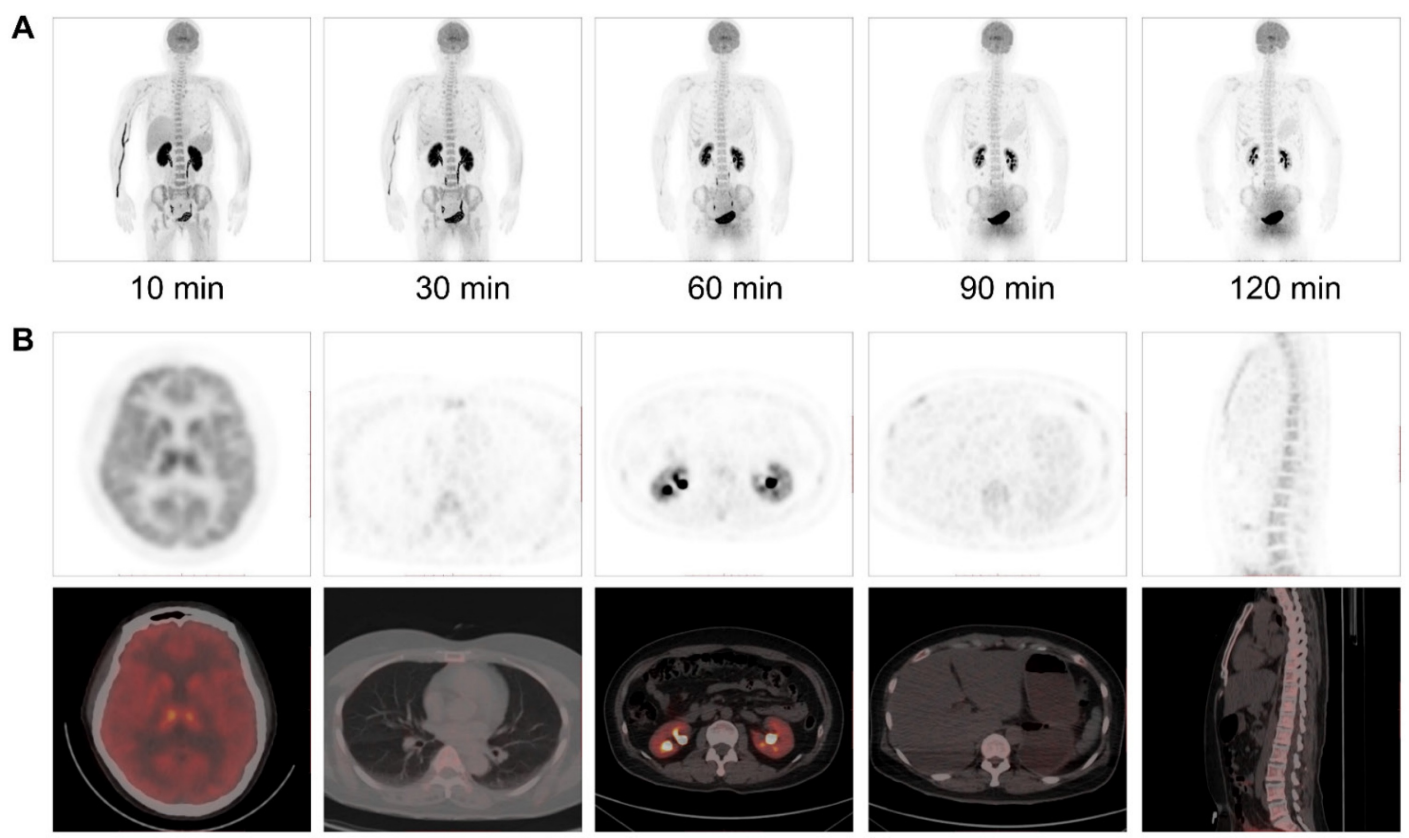
(A) Multiple time-point whole-body maximum intensity projection (MIP) PET imaging was performed on a 52-year-old female volunteer at 10, 30, 60, 90 and 120 min after intravenous administration of [^18^F]DMPY2. (B) PET/CT imaging at 60 min post-injection demonstrated radiotracer uptake in major organs.

**Figure 2:**
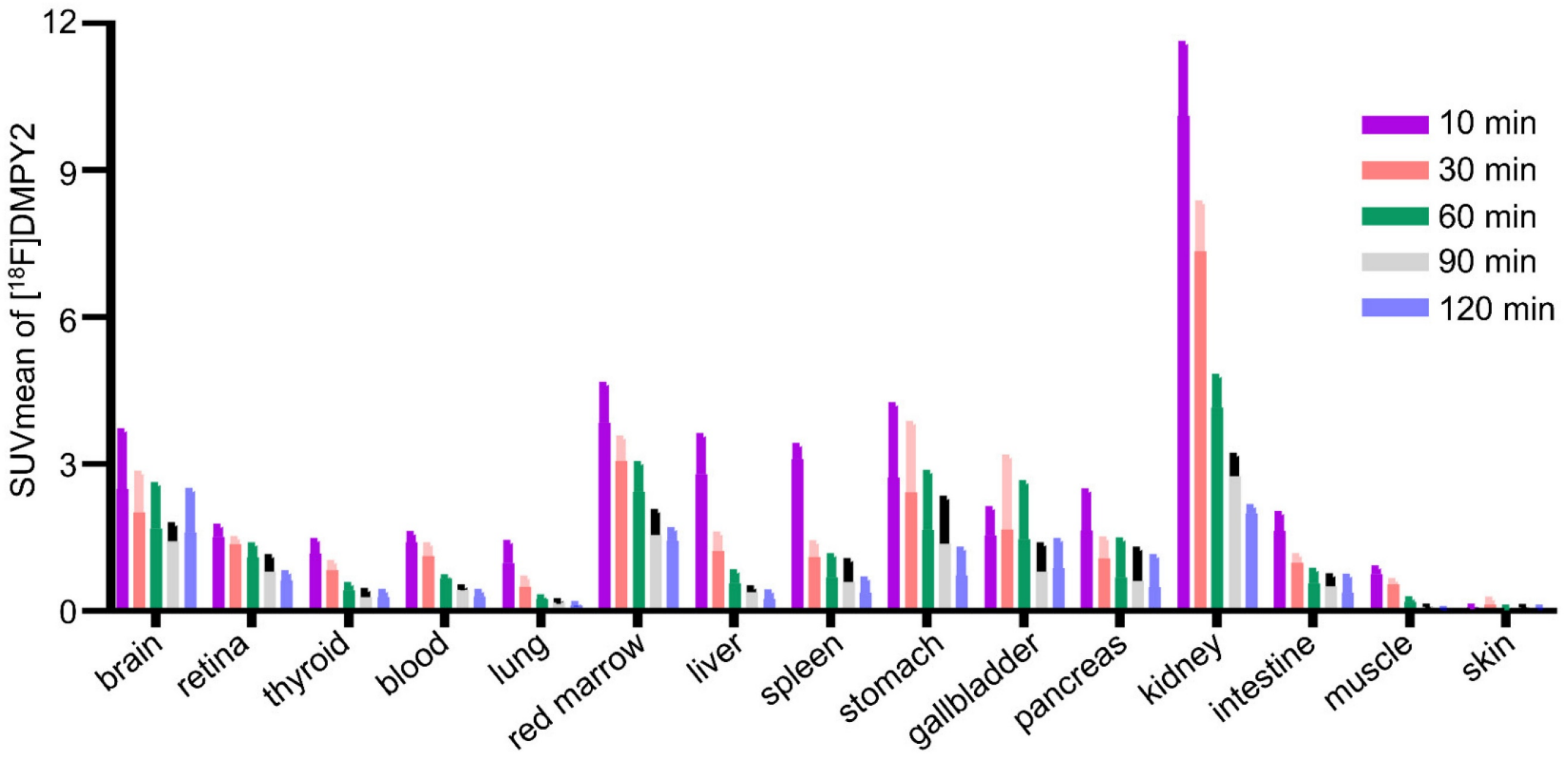
SUVmean of [^18^F]DMPY2 uptakes in various organs was quantified at multiple time points post-intravenous administration in human volunteers.

The biodistribution of [^18^F]DMPY2 in a healthy volunteer is presented in Figure 1 at 10, 30, 60, 90, and 120 min after intravenous [^18^F]DMPY2 injection. SUV(mean) decreased in most organs at various time points as shown in Figure 2, including kidney, liver, spleen, stomach, lung, and muscle. Notably, the kidneys exhibited high uptake and rapid reduction, indicating quick clearance of [^18^F]DMPY2 via the urinary system. Physiological uptake was observed in the retina, stomach, and gallbladder, with 60-minute SUVmean values ranging from 1.09 to 1.65. Moderate uptake was seen in the lungs, liver, and brain at 60 min post-injection, with SUVmean values of 0.24 ± 0.02, 0.57 ± 0.12, and 1.69 ± 0.51, respectively. The skin showed low SUVmean values at 10, 30, 60, 90, and 120 min, recorded at 0.07 ± 0.01, 0.14 ± 0.06, 0.07 ± 0.004, 0.07 ± 0.01, and 0.05 ± 0.01, respectively. This low uptake is promising for effectively visualizing primary cutaneous lesions.

The estimated absorbed dose of [^18^F]DPMY2 for various organs is shown in Table 2. The calculated average systemic effective dose (ED) was 0.012 ± 0.001 mSv/MBq. High-dose activities were observed in the urinary bladder wall (0.017 ± 0.006 mSv/MBq), ovaries (0.019 mSv/MBq), osteogenic cells (0.21 ± 0.004 mSv/MBq), prostate (0.018 mSv/MBq), red marrow (0.26 ± 0.007 mSv/MBq), and left colon (0.018 ± 0.001 mSv/MBq).

### Clinical characteristics of MM patients

Thirty-one patients with pathologically confirmed MM were enrolled in the study, with clinical characteristics summarized in Table 3. Basic information included patient demographics, the clinical role of PET/CT imaging, pathological results from Hematoxylin and Eosin (HE), immunohistochemistry (IHC) staining, clinical stage, examination type, treatment management, and numbers of LN biopsy and metastatic LN. Twenty-five MM patients underwent initial staging examinations, and five patients had already undergone primary cutaneous tumor removal at another hospital, subsequently receiving LN dissection. Only one patient required restaging the after the disease recurrence, with subsequent surgical intervention planned. In addition, LN biopsies were conducted in twenty-seven patients based on clinical indications, with postoperative pathology compared to PET uptake. Most primary lesions were in lower limb skin, except for one patient with gingival mucosa MM. Over half of MM patients were diagnosed as stage І or ІІ and the remainder had resectable disease according to clinical guidelines.

### Primary lesion analysis

**Figure 3:**
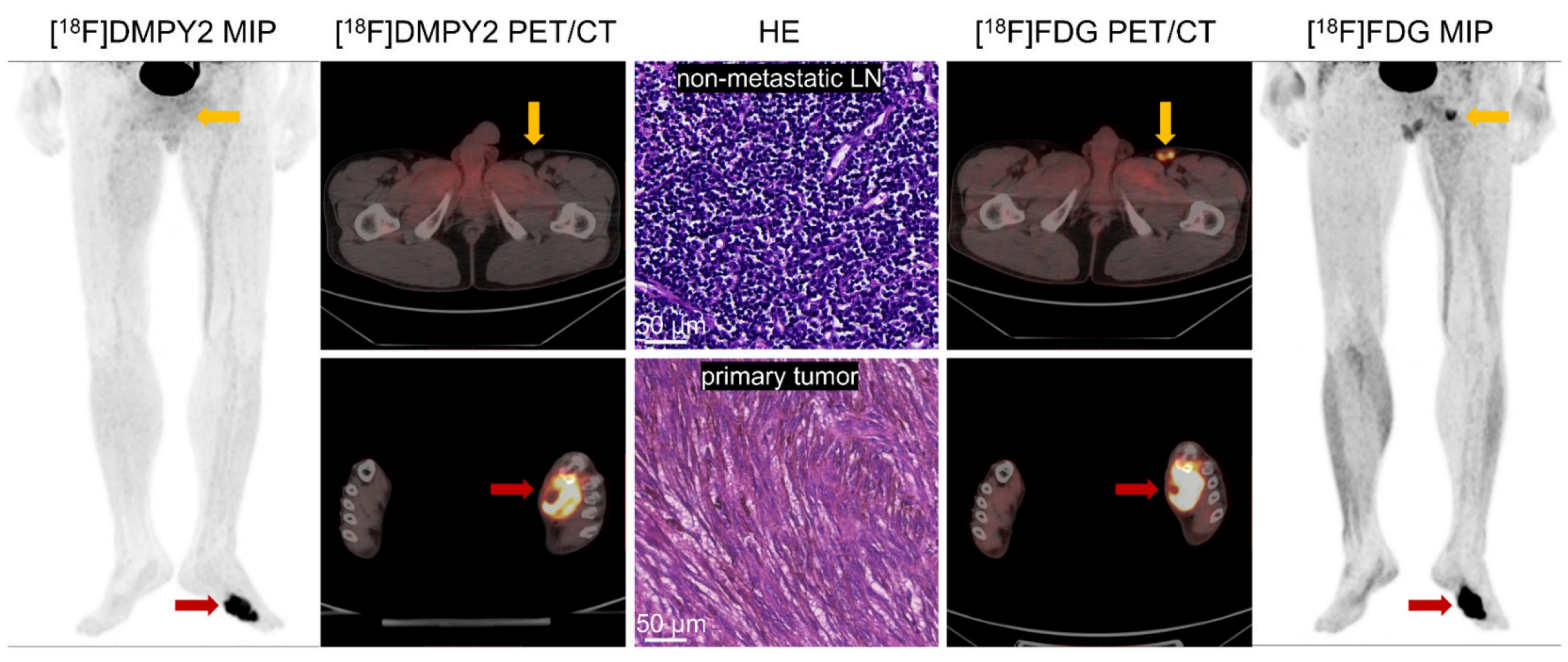
Local recurrence of the left plantar skin in postoperative MM patient #10 was detected by [^18^F]FDG and [^18^F]DMPY2 PET/CT. Both tracers demonstrated high uptake in the left plantar skin recurrence, with [^18^F]DMPY2 and [^18^F]FDG PET/CT yielding raw SUVmax values of 9.94 and 13.69, respectively. The left inguinal lymph node exhibited no significant uptake on [^18^F]DMPY2 PET/CT and was confirmed to be non-metastatic by pathology (no evidence of malignant cells confirmed by HE staining). In contrast, it showed high uptake (raw SUVmax 5.94) on [^18^F]FDG PET/CT.

**Figure 4:**
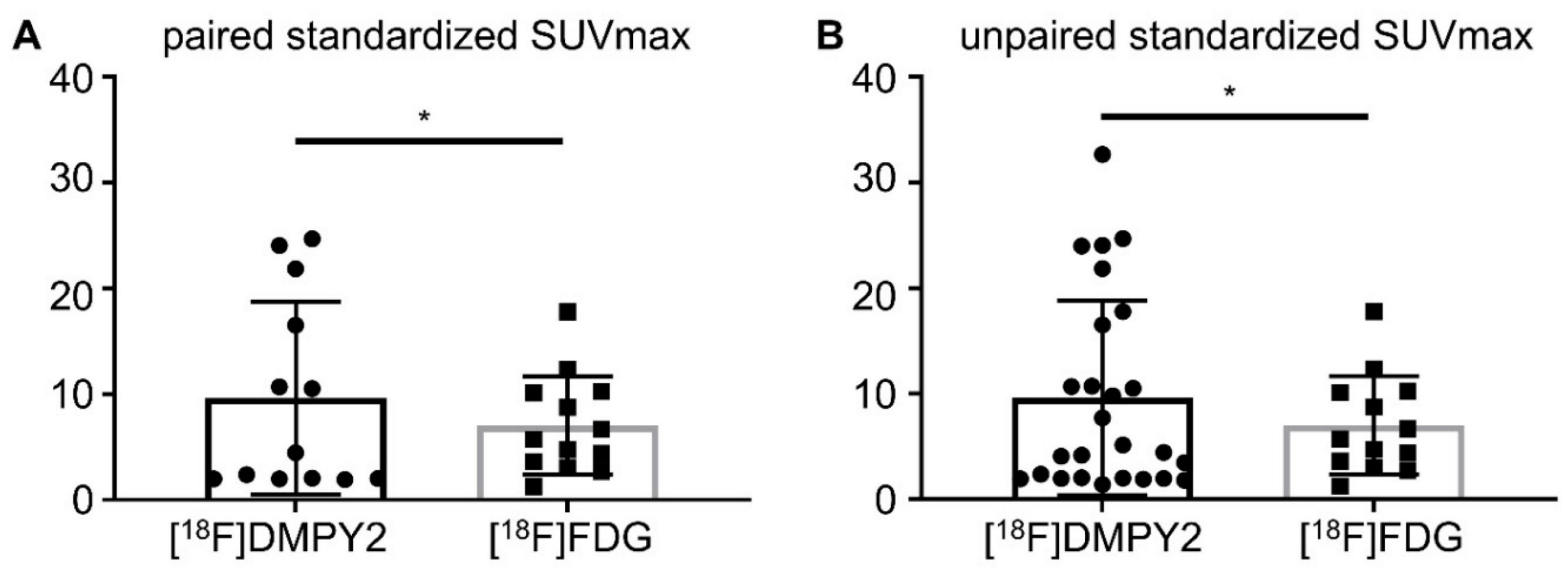
Analysis of Paired Standardized SUVmax (A) and Unpaired Standardized SUVmax (B) of MM primary tumors by [^18^F]DMPY2 and [^18^F]FDG PET/CT.

Like [^18^F]FDG PET/CT, [^18^F]DMPY2 PET/CT successfully identified all primary neoplastic lesions. Both tracers demonstrated high uptake and a favorable tumor-to-background ratio in primary tumor lesions (Figure 3). Figure S2 showed that the SUVmax of [^18^F]DMPY2 was significantly lower than that of [^18^F]FDG. However, in paired standardized SUVmax analysis (shown in Figure 4), the DMPY2 value (9.62 ± 2.54, n = 13) was significantly higher than the FDG value (7.03 ± 1.29, n = 13; p = 0.027). In paired Standardized SUVmax analysis with combination of single DMPY2 imaging, DMPY2 uptake was also superior to FDG uptake (9.61 ± 1.81, n = 26 vs. 7.03 ± 1.29, n = 13; p = 0.017).

### Assessments based on drainage LN

A total of twenty-seven MM patients and fifty-one resected LN confirmed by pathology were evaluated to assess the diagnostic performance of [^18^F]DMPY2. Among them, fifteen patients underwent both [^18^F]DMPY2 and [^18^F]FDG PET/CT scans, revealing eight positive and seven negative LN biopsies. Additionally, eleven patients underwent [^18^F]DMPY2 PET imaging alone, with one case showing lymph node metastasis and eleven cases showing non-metastatic lymph node involvement.

In patient-based analysis, among all patients who underwent [^18^F]DMPY2 imaging, there were nine metastatic lymph nodes (with six true positives and three false negatives) and eighteen non-metastatic lymph nodes (with eighteen true negatives and zero false positives). In [^18^F]FDG imaging, there were eight metastatic lymph nodes (with four true positives and four false negatives) and seven non-metastatic lymph nodes (with three true negatives and four false positives). In LN-based analysis, [^18^F]DMPY2 imaging identified fifteen metastatic lymph nodes (eight true positives and seven false negatives) and thirty-six non-metastatic lymph nodes (thirty-six true negatives and zero false positives). In contrast, [^18^F]FDG imaging detected fourteen metastatic lymph nodes (six true positives and eight false negatives) and nineteen non-metastatic lymph nodes (twelve true negatives and seven false positives). Thus, [^18^F]DMPY2 demonstrated superior diagnostic performance over [^18^F]FDG in both patient-based and LN-based analyses (Table 4). In patient-based analysis, the sensitivity, specificity, accuracy, and positive and negative predictive values of [^18^F]DMPY2 were higher compared to [^18^F]FDG (66.7% vs. 50%, 100% vs. 42.9%, 88.9% vs. 46.7%, 100% vs. 50%, 85.7% vs. 42.9%, respectively). In LN-based analysis, the sensitivity, specificity, accuracy, and positive and negative predictive values of [^18^F]DMPY2 were higher compared to [^18^F]FDG (53.3% vs. 42.9%, 100% vs. 63.2%, 86.3% vs. 54.5%, 100% vs. 46.2%, 83.7% vs. 60%, respectively). The ROC curves confirmed significant differences in assessing LN metastases between [^18^F]DMPY2 and [^18^F]FDG PET/CT (AUC of DMPY2: 0.922 ± 0.079 vs. AUC of FDG: 0.675 ± 0.133; p = 0.036) in patient-based analysis. In the LN-based analysis, the p-value between the AUC of DMPY2 and FDG difference was 0.0018, as shown in Figure S3. As shown in Figure S4, [^18^F]DMPY2 Standardized SUVmax in metastatic LN (6.352 ± 2.017) was significantly higher compared to non-metastatic LN (0.750 ± 0.059) and confirmed by pathology (p < 0.0001).

**Figure 5:**
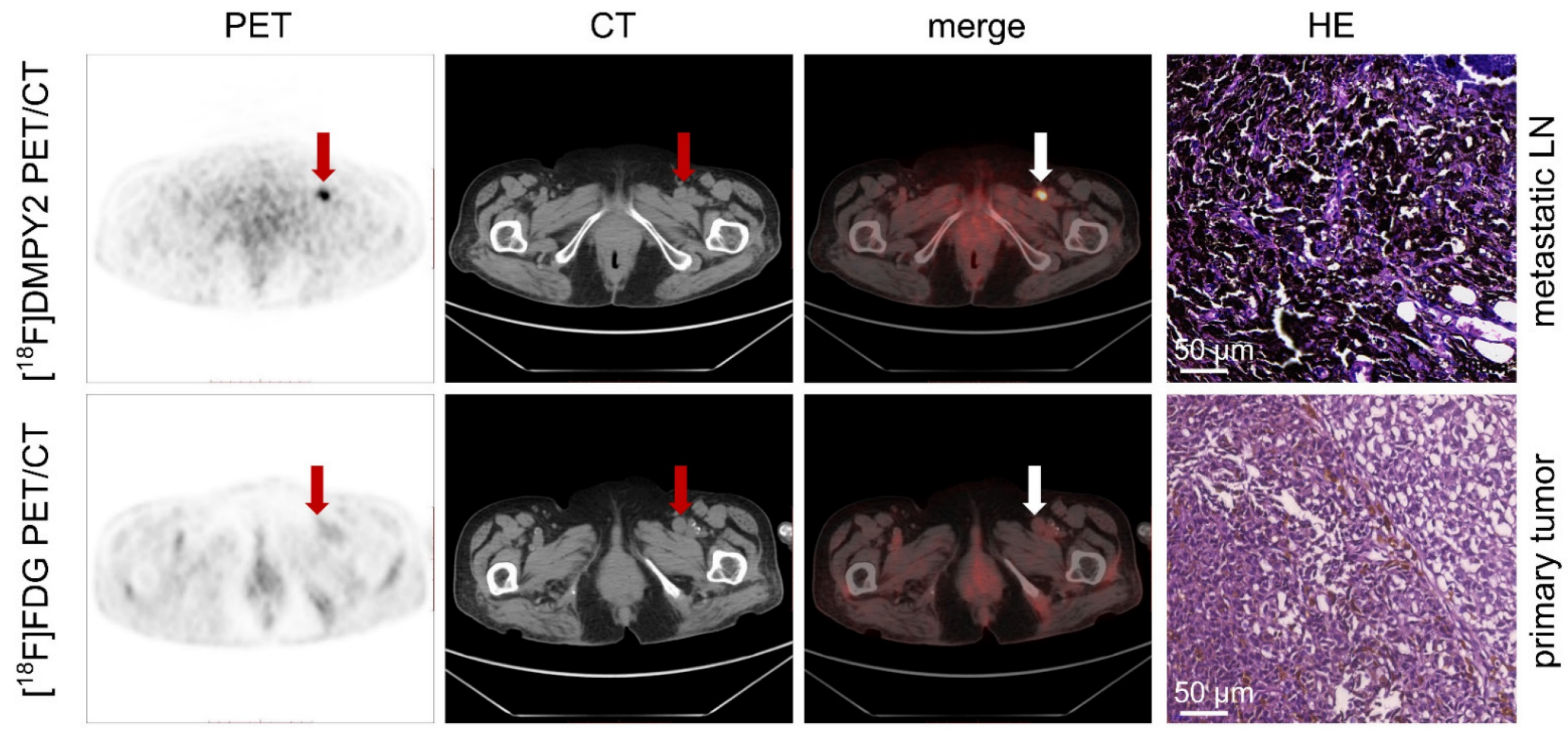
Enlarged left inguinal lymph was detected in MM patient #5 by[^18^F]FDG and [^18^F]DMPY2 PET/CT. High uptake (raw SUVmax 9.23) of the left inguinal lymph node, which was confirmed positive (LN IHC: S-100 +++, Melan-A ++, HMB45 ++) by pathology, in [^18^F]DMPY2 PET/CT while low uptake (raw SUVmax 2.99) in [^18^F]FDG PET/CT. HE staining of the metastatic lymph node is also shown.

In a case study presented in Figure 3, a patient with local recurrence of left plantar skin was identified using [^18^F]FDG and [^18^F]DMPY2 PET/CT, showing high uptakes (SUVmax 9.94 vs. 13.69). The absence of [^18^F]DMPY2 uptake in left inguinal LN, confirmed as negative by histopathology, contrasted with significant uptake (raw SUVmax 5.94) on [^18^F]FDG PET/CT. Similar findings in Figure S5 highlighted [^18^F]DMPY2's accuracy in detecting non-metastatic LN, whereas [^18^F]FDG PET identified a false-positive lesion. Figure 5 displays a patient with pathologically confirmed LN metastasis (LN IHC: S-100 +++, Melan-A ++, HMB45 ++) demonstrated high [^18^F]DMPY2 uptake (raw SUVmax 9.23) as a true-positive result, contrasting with low [^18^F]FDG uptake (raw SUVmax 2.99) indicating a false-negative finding. Overall, [^18^F]DMPY2 demonstrated significantly superior diagnostic performance to [^18^F]FDG in distinguishing metastatic lymph nodes among surgical MM patients.

### Advantages of [^18^F]DMPY2 in imprecise lesions

[^18^F]DMPY2, as a melanoma-specific imaging agent, may provide significant advantages by overcoming the limitations of [^18^F]FDG, distinguishing melanoma from inflammatory lesions and other tumors, and reducing false-positive results. In the seventeen patients who underwent [^18^F]DMPY2 and [^18^F]FDG imaging, excluding the draining lymph node assessment, a total of seven [^18^F]FDG-positive lesions were identified that were difficult to differentiate by two experienced nuclear medicine physicians. These lesions were subsequently confirmed to be non-malignant by follow-up, including fungal infections, benign bone lesions, dentoalveolar abscess, and pulmonary adenocarcinoma. Among these seven lesions, [^18^F]DMPY2 showed moderate uptake in one benign bone lesion, while the remaining six lesions exhibited negligible uptake. The raw SUVmax values of [^18^F]FDG (5.26 ± 0.70, n = 7) and [^18^F]DMPY2 (1.28 ± 0.37, n = 7) in these lesions were statistically significantly different (p = 0.0003).

**Figure 6:**
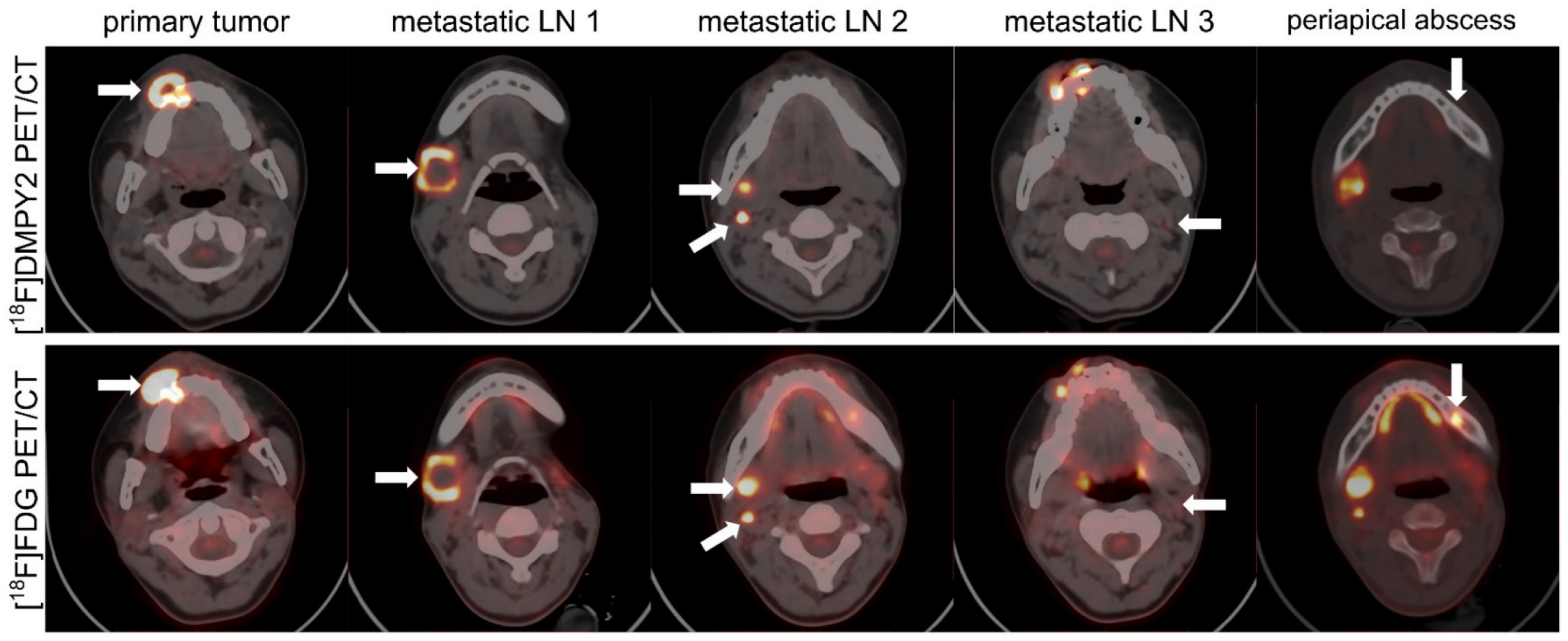
Lymph node and bone metastases in a 17-year-old female MM patient #14 were detected by [^18^F]FDG and [^18^F]DMPY2 PET/CT. White arrows indicate regions of significant tracer uptake. The primary lesion invaded the right upper alveolar bone, causing osteolytic destruction. A left upper alveolar lesion showed high [^18^F]FDG uptake but no [^18^F]DMPY2 and was diagnosed as a periapical abscess. In [^18^F]DMPY2 PET, two hypermetabolic foci in the right neck level II LN and uptake in the left neck level II LN were histopathologically confirmed as metastatic, while the left neck LN appeared ambiguous on [^18^F]FDG PET.

Patient #14, a 17-year-old female with melanoma, presented a progressing pigmented lesion on the right upper gingival mucosa (Figure 6), which invaded the right upper alveolar bone, resulting in osteolytic bone destruction. A confounding lesion in the left upper alveolar bone, characterized by high [^18^F]FDG uptake but no [^18^F]DMPY2 uptake, was clinically confirmed as a periapical abscess. And in [^18^F]DMPY2 PET imaging, two hypermetabolic foci in the right neck level ІІ LN and a clear uptake in left neck level ІІ LN in [^18^F]DMPY2 PET were confirmed pathologically as metastatic involvement. However, the left neck level II LN posed diagnostic ambiguity on [^18^F]FDG PET, where visual analysis could not reliably differentiate between inflammatory hyperplasia and metastatic infiltration.

**Figure 7:**
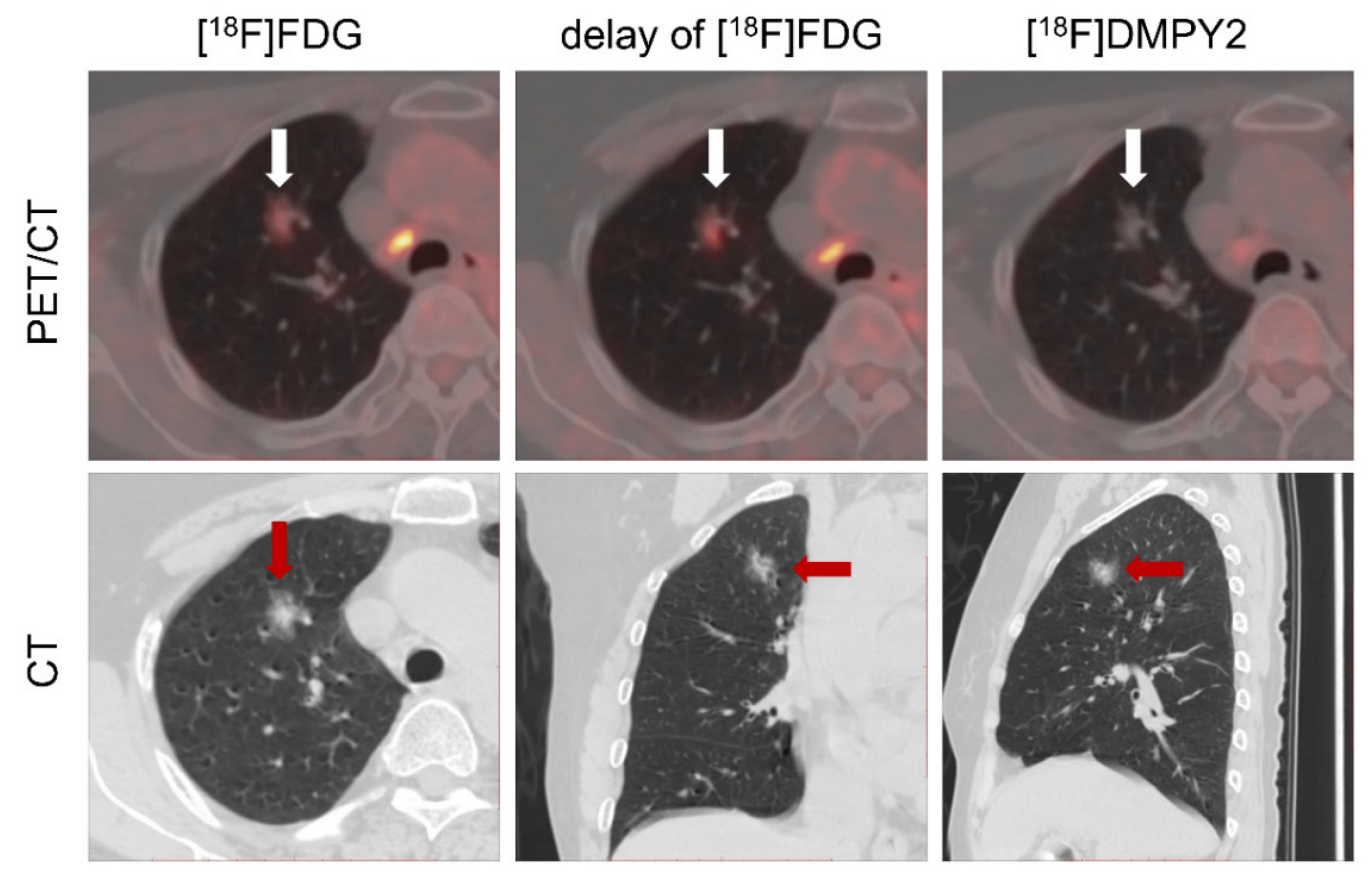
LN metastases and a suspicious lung nodule in MM patient #16 were detected by [^18^F]FDG and [^18^F]DMPY2 PET/CT. The lung nodule exhibited a 46% increase in delayed phase [^18^F]FDG uptake (raw SUVmax 3.75 vs. 2.56). However, no uptake was observed on [^18^F]DMPY2 PET/CT, suggesting a non-melanoma etiology.

Patient #13 (Figure S5) demonstrated a well-defined [^18^F]DMPY2 signal at the right lateral margin of the big toe, whereas [^18^F]FDG imaging exhibited confounding uptake due to coexisting tinea pedis in the right abdominal and lateral toe regions. This observation underscored [^18^F]DMPY2's reduced susceptibility to inflammatory interference and low false-positive rates, particularly relevant in Asian populations with a high prevalence of acral-type melanoma. In Patient #16 shown in Figure 7, referred for melanoma restaging post-resection, a suspicious lung nodule in the apical segment of the right superior lobe displayed moderate FDG uptake (SUVmax 2.56) with a 46% increase on delayed imaging (SUVmax 3.75), suggestive of neoplastic activity. However, [^18^F]DMPY2 PET/CT revealed no tracer accumulation in the lesion. Integrated with clinical indices and medical history, the nodule was conclusively diagnosed as primary lung carcinoma rather than melanoma metastasis.

In summary, [^18^F]DMPY2 PET/CT can clearly delineate the lesions and provide useful information to differentiate inflammatory and neoplastic lesions.

## Discussion

In this research study, [^18^F]DMPY2 exhibits favorable pharmacokinetic characteristics, minimal liver background, and fast renal clearance, making it a safe and well-tolerated melanin PET tracer for clinical imaging. In addition, the clinical application of [^18^F]DMPY2 was illustrated. First, notable [^18^F]DMPY2 uptake within the tumor and elevated tumor-to-background ratios in primary MM lesions were observed in contrast to [^18^F]FDG. Second, the diagnostic efficacy of [^18^F]DMPY2 surpassed that of [^18^F]FDG in detecting LN metastases in surgical MM patients. Furthermore, [^18^F]DMPY2 PET imaging had a unique advantage in distinguishing [^18^F]FDG false-positive lesions of MM patients. With a high positive predictive value for LN metastasis, superior to other imaging modalities, [^18^F]DMPY2 PET/CT can guide surgical decisions regarding LN biopsy or dissection in MM patients, crucial for optimizing treatment outcomes amidst high recurrence risks and higher LN metastatic burdens across Asian populations. In some cases, LN metastases were not successfully visualized by either [^18^F]DMPY2 or [^18^F]FDG PET/CT. Upon reviewing the pathological findings of these melanoma patients, we observed that the metastatic lymph nodes contained only small tumor nests, suggesting that the tumor burden may have been below the detection threshold of PET imaging 18. This limitation is inherent to molecular imaging techniques, as PET resolution may not always allow for the detection of microscopic metastases. A more comprehensive integration of PET/CT parameters, and clinical and pathological data may optimize the sensitivity of LN metastasis. Its gallbladder and gastrointestinal tract absorption, possibly due to partial hepatobiliary excretion and mucosal melanin distribution, further underpin its biodistribution characteristics 19. Due to the limited melanin presence in the central nervous system, [18F] DMPY2 is taken up in the brain in negligible amounts, suggesting its potential for detecting brain metastases requires further investigation.

Ma et al. and Zhang et al. have conducted clinical research using melanin-targeted PET tracers, [^18^F]P3BZA and [^18^F]PFPN 15, 16. As for radiation dosimetry, [^18^F]DMPY2 demonstrated the lowest total effective dose (0.012 mSv/MBq), outperforming [^18^F]P3BZA (0.0193 mSv/MBq) and [^18^F]PFPN (0.0201 mSv/MBq), the latter being comparable to [^18^F]FDG (0.02 mSv/MBq) 20. All three probes exhibited excellent safety and tolerability profiles and achieved a 100% detection rate for melanoma primary lesions. However, the small sample size of [^18^F]P3BZA (n = 5) necessitates further validation. For metastatic lesion detection, [^18^F]P3BZA PET/CT and [^18^F]FDG PET/CT both identified two lymph node metastases and 1 bone metastasis. [^18^F]PFPN PET/MRI, evaluated in twenty-one advanced melanoma patients, outperformed [^18^F]FDG PET/CT in metastatic lesion detection.

In contrast, our study included thirty-one surgical melanoma patients with pathological confirmation, demonstrating that [^18^F]DMPY2 PET/CT achieved exceptional specificity and positive predictive value (both 100%) for lymph node metastasis prediction, significantly surpassing [^18^F]FDG PET/CT, making it suitable for preoperative assessment and postoperative monitoring. Additionally, [^18^F]DMPY2 showed superior performance in distinguishing false-positive lesions compared to [^18^F]FDG, while data on [^18^F]P3BZA and [^18^F]PFPN in this respect remain limited. Thus, [^18^F]DMPY2 offers distinct advantages in radiation dose efficiency, lymph node metastasis prediction, and false-positive lesion differentiation.

In MM patients, melanin can regulate epidermal homeostasis, thus affecting melanoma progression and therapeutic outcomes 21. Studies have shown that increased melanin production is associated with reduced overall survival and disease-free survival in patients with metastatic melanoma 22. PET imaging is a valuable tool for assessing treatment response in MM patients 23, and [^18^F]DMPY2 PET has the potential to serve as a novel imaging modality for evaluating the effectiveness of systemic therapies. However, the imaging performance of [^18^F]DMPY2 PET may not be as good as that of [^18^F]FDG PET in non-pigmented MM , as its uptake depends on melanin expression. Melanin exhibits paramagnetic properties due to its free radicals, which contain unpaired electrons and hydrogen ions, resulting in high signal intensity on T1-weighted MRI, providing a significant advantage in signal resolution 24. Consequently, melanin-targeted [^18^F]DMPY2 PET combined with MRI could enable truly effective multi-mode fusion imaging, enhancing diagnostic accuracy and lesion characterization. Moreover, [^18^F]DMPY2, as an ideal melanin-targeting tracer, can improve the accuracy of melanoma diagnosis and possibly serve as a molecular platform for targeted radionuclide therapy 25. This theranostic approach would facilitate personalized treatment strategies, allow real-time monitoring of therapeutic response via PET imaging and optimize patient management.

## Conclusion

In this study, [^18^F]DMPY2, a safe and well-tolerated PET tracer, demonstrated significant clinical value in the early detection of MM primary lesions and diagnostic evaluation of LN metastases. It is a pivotal tool for optimized staging and therapeutic decision-making, thereby enhancing patient prognosis.

## Supplementary Material

Supplementary figures and tables.

## Figures and Tables

**Table 1:** Summary of volunteer characteristics.

Characteristics	Value
No. of Volunteer	3
**Age(year)**	
Median	37
Interquartile range	36-41
**Sex**	
Men	2
Women	1

**Table 2:** Estimated absorbed radiation dosimetry for [^18^F]DMPY2 in different human organs.

Organ	Dose (mSv/MBq)
Adrenals	1.53E-02
Brain	8.43E-03
Esophagus	1.33E-02
Eyes	1.37E-02
Gallbladder Wall	1.51E-02
Left colon	1.78E-02
Small Intestine	1.74E-02
Stomach Wall	1.46E-02
Right colon	1.78E-02
Rectum	1.85E-02
Heart Wall	6.85E-03
Kidneys	8.55E-03
Liver	6.33E-03
Lungs	7.90E-03
Pancreas	1.35E-02
Uterus (n = 1)	1.45E-02
Prostate (n = 2)	1.83E-02
Salivary Glands	1.53E-02
Red Marrow	2.57E-02
Osteogenic Cells	2.13E-02
Spleen	1.14E-02
Breasts (n = 1)	4.95E-03
Ovaries (n = 1)	1.90E-02
Testes (n = 2)	8.86E-03
Thymus	1.42E-02
Thyroid	1.51E-02
Urinary Bladder Wall	1.74E-02
Total Body	1.56E-02
Effective Dose	1.22E-02

**Table 3:** Summary of patient characteristics.

Patient number	Weight (kg)	Age (year)	Examination (PET/CT)	Treatment	Number of LN biospy	Number of metastatic LN
1	62	62	DMPY2+FDG	Surgery+SLNB	1	1
2	65	65	DMPY2+FDG	Surgery+SLNB	2	2
3	60	17	DMPY2+FDG	Surgery+SLNB	2	2
4	54	32	DMPY2+FDG	Surgery+SLNB	2	0
5	49	74	DMPY2+FDG	Surgery+SLNB	1	1
6	55	56	DMPY2+FDG	Surgery+SLNB	2	0
7	56	78	DMPY2+FDG	Surgery+SLNB	4	2
8	46	80	DMPY2+FDG	Surgery+SLNB	2	1
9	68	53	DMPY2+FDG	Surgery+SLNB	2	0
10	60	53	DMPY2+FDG	Surgery+SLNB	2	0
11	70.5	40	DMPY2+FDG	Surgery+SLNB	5	2
12	61	67	DMPY2+FDG	Surgery+SLNB	2	0
13	61	56	DMPY2+FDG	Surgery+SLNB	2	0
14	45	17	DMPY2+FDG	Surgery+SLNB	3	3
15	45	45	DMPY2+FDG	Surgery	0	0
16	54	69	DMPY2+FDG	Surgery	0	0
17	49	62	DMPY2+FDG	Surgery	1	0
18	61	58	DMPY2	Surgery+SLNB	1	0
19	45	47	DMPY2	Surgery+SLNB	1	0
20	87	69	DMPY2	Surgery+SLNB	1	0
21	60	51	DMPY2	Surgery+SLNB	1	0
22	57	65	DMPY2	Surgery+SLNB	1	0
23	50	63	DMPY2	Surgery+SLNB	2	0
24	65	63	DMPY2	Surgery+SLNB	1	0
25	56	51	DMPY2	Surgery+SLNB	1	0
26	45	80	DMPY2	Surgery+SLNB	1	0
27	61	63	DMPY2	Surgery+SLNB	1	0
28	75	57	DMPY2	Surgery+SLNB	5	1
29	65	56	DMPY2	Surgery	0	0
30	53	53	DMPY2	Surgery+SLNB	2	0
31	45	87	DMPY2	Surgery	0	0

Abbreviations: SLNB, sentinel lymph node biopsy.

**Table 4:** Diagnostic performance of [^18^F]DMPY2 and [^18^F]FDG PET/CT in assessment of lymph node metastases in patient-based analysis and lesion-based analysis

Analysis	Modality	SE	SP	PPV	NPV	ACC
Patient	DMPY2	66.7 (6/9)	100 (18/18)	100 (6/6)	85.7 (18/21)	88.9 (24/27)
	FDG	50 (4/8)	42.9 (3/7)	50 (4/8)	42.9 (3/7)	46.7 (7/15)
Lesion	DMPY2	53.3 (8/15)	100 (36/36)	100 (8/8)	83.7 (36/43)	86.3 (44/51)
	FDG	42.9 (6/14)	63.2 (12/19)	46.2 (6/13)	60 (12/20)	54.5 (18/33)

Abbreviations: Se for sensitivity, Sp for specificity, PPV for positive predictive value, NPV for negative predictive value and ACC for accuracy.

## Abbreviations

Acc: accuracy
CT: computed tomography
[^18^F]DMPY2: N-(2-(dimethylamino) ethyl)-5-[^18^F] fluoropicolinamide
[^18^F]FDG: [^18^F]-fluorodeoxyglucose
GE: general electric
LN: lymph node
MM: malignant melanoma
MRI: magnetic resonance imaging
NPV: negative predictive value
PET: positron emission tomography
PPV: positive predictive value
ROI: region of interest
Se: sensitivity, Sp: Specificity
SLNB: Sentinel lymph node biopsy
SUVmax: maximum standardized uptake value
SUVmean: mean standardized uptake value
