# Supplementary material for: First-in-human PET imaging and evaluation of melanin-targeted [18F]DMPY2 in malignant melanoma patients
Source: Theranostics. 2025 May 7;15(13):6100–10. doi: 10.7150/thno.109243 (PMC12159754; doi:10.7150/thno.109243)
Supplement: Supplementary file 1 — Supplementary figures and tables. [file thnov15p6100s1.pdf]

Supplementary data

Synthesis and analysis of [^{18}F]DMPY2

[^{18}F]Fluoride was produced by a cyclotron (GE Qilintrace, USA) by irradiating [^{18}O]H_2O via a (p,n) reaction for 1 h. The [^{18}F]fluoride was transferred to a hot cell containing a synthesis module (GE Tracerlab FXFN,USA) for automated radiosynthesis. The [^{18}F]fluoride was trapped on a quaternary methyl ammonium (QMA) cartridge(Waters Corporation, USA) and the activity was eluted into the reaction vial with a solution of Kryptofix 222 (19.1 mg) and potassium bicarbonate (3.8 mg) in acetonitrile and water (85%/15% v/v; 1.0 mL). The eluted mixture was dried via azeotropic distillation by heating the reaction vial to 110 °C under N_2-flow with 2 additions of MeCN over 20 min. The chemical precursor (5 mg) dissolved in dry DMSO (0.7 mL) was added to the dried [^{18}F]fluoride mixture and the reaction was heated to 120 ℃ for 15 min. The reaction was cooled and then quenched by addition of 3 mL of H_2O. The diluted reaction crude was injected on a semi-preparative HPLC column (Luna C18 semipreparative column (10 × 250 mm, 10 µm, Phenomenex, Torrance, CA, USA) with an eluent consisting of water and MeCN 80%/20% v/v, each containing 0.5% trifluoroacetic acid), at a flow rate of 3 mL/min. The fraction corresponding to the desired product was collected and diluted by 45 mL H_2O. The diluted fraction was extracted using a C18-cartridge (Waters Corporation, USA). The C18-cartridge was washed with 10 mL of H_2O and then eluted using 2.0 mL of EtOH into a transfer vial containing 20 mL saline solution. The complete content was subsequently transferred to the final product via a sterile filter (Merck Millipore, USA).

Quality control was performed using Agilent 1260 Infinity II HPLC (Palo Alto, USA) with a Bioscan flow-count radioactivity detector and a ZORBAX SB-C18 column (5 µm 4.6 × 250 mm). The HPLC was eluted with water-acetonitrile system (Phase A: 0.1 % TFA + H_2O; Phase B: 0.1 % TFA + CH_3CN) using gradient elution (0-2 min 5 % B; 2-15 min 5 %-90 % B) at a flow rate of 2.0 mL min^{-1}.

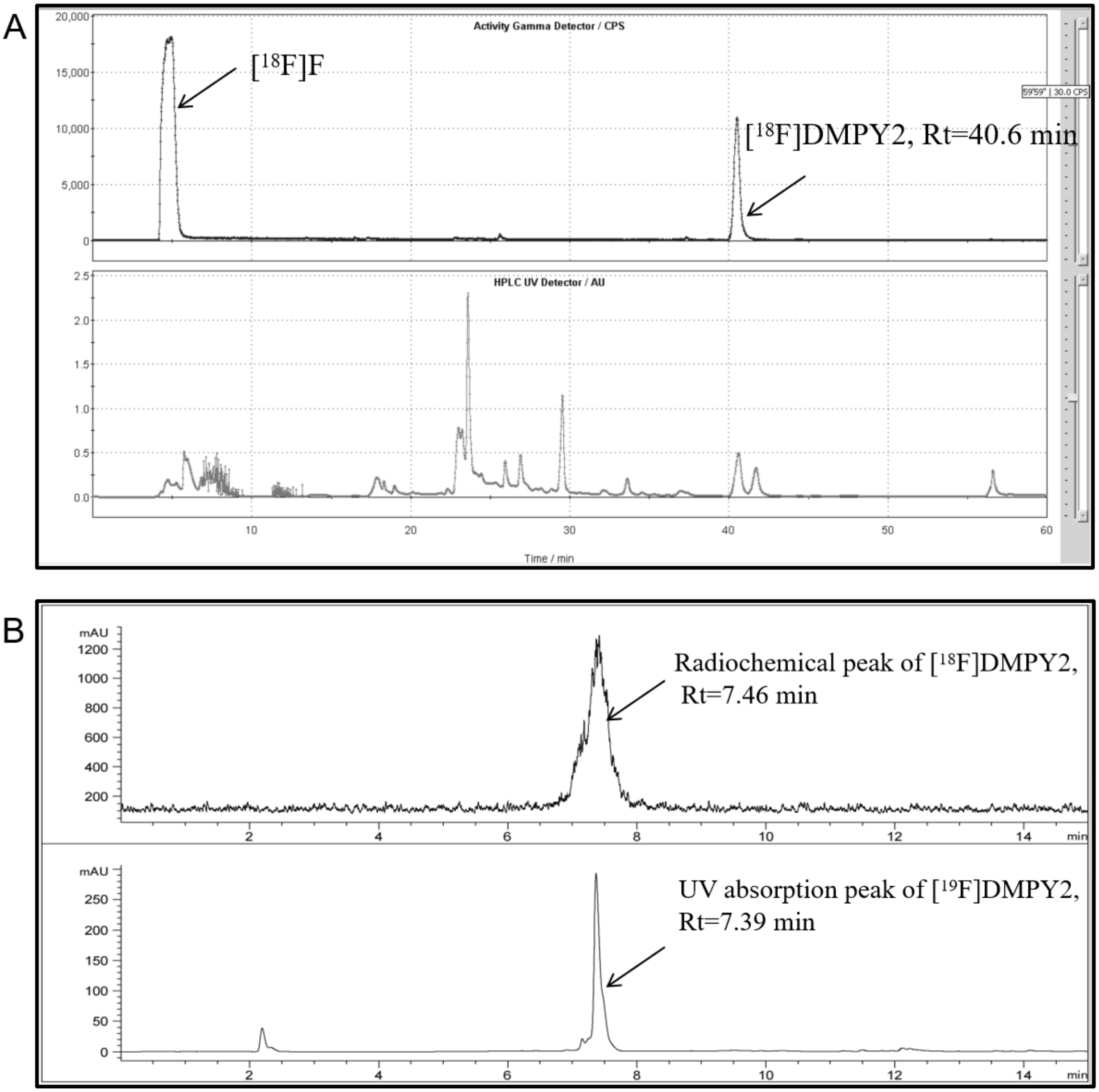

Supplemental Figure 1. The radio semi-preparative HPLC (A) and analytic HPLC (B) of [^{18}F]DMPY2.

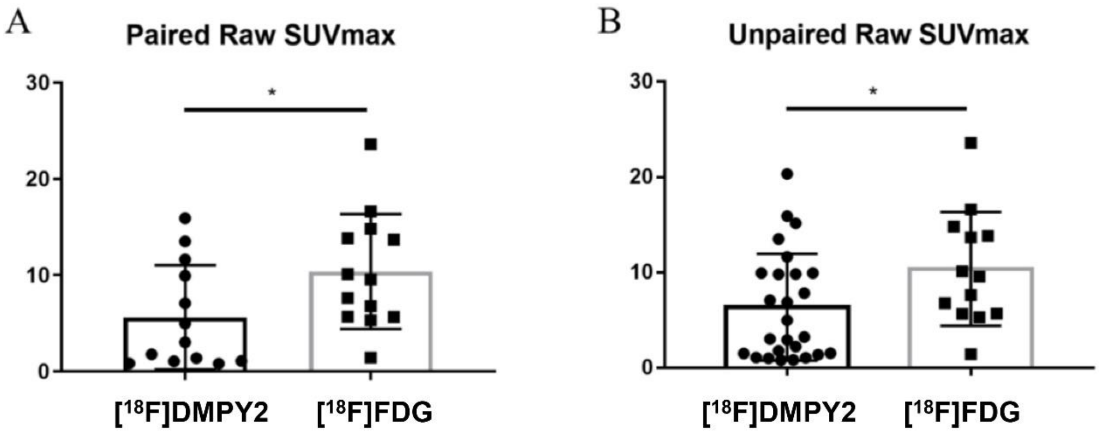

Supplemental Figure 2. The analysis of Paired Raw SUVmax (A) and Unpaired Raw SUVmax (B) of MM primary lesions tumors between [^{18}F]DMPY2 and [^{18}F]FDG PET/CT.

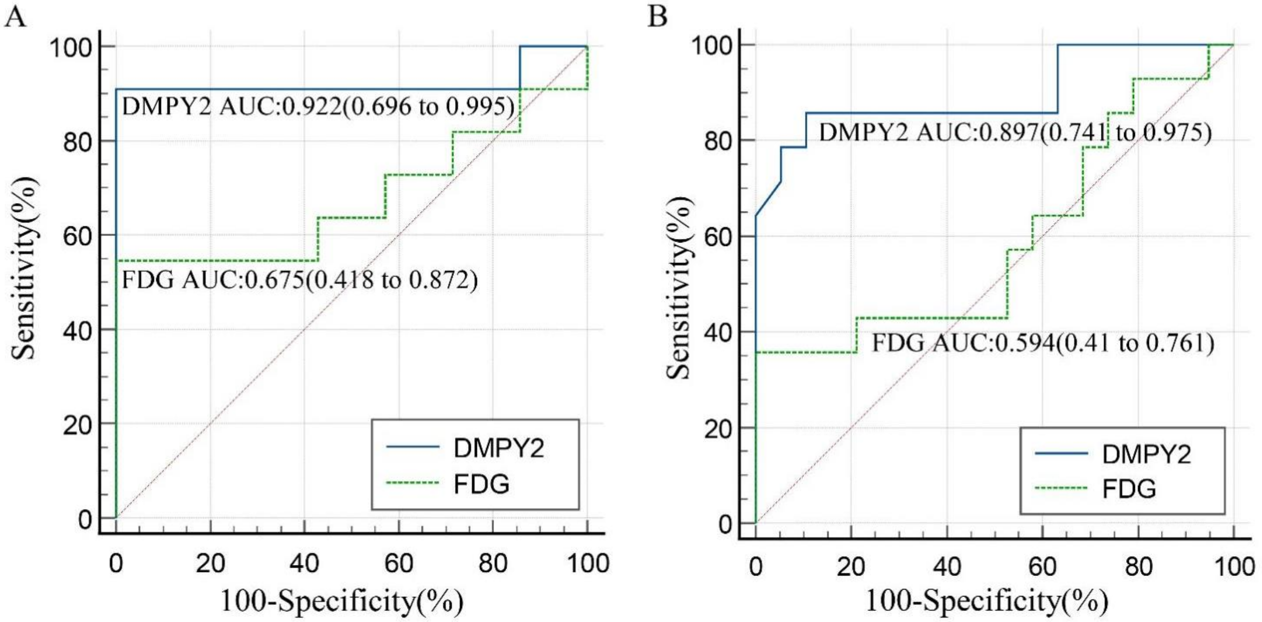

Supplemental Figure 3 Comparison of independent ROC curves between [^{18}F]DMPY2 and [^{18}F]FDG PET/CT in Assessment of Lymph Node Metastases in patient-based analysis(A) and LN-based analysis(B).

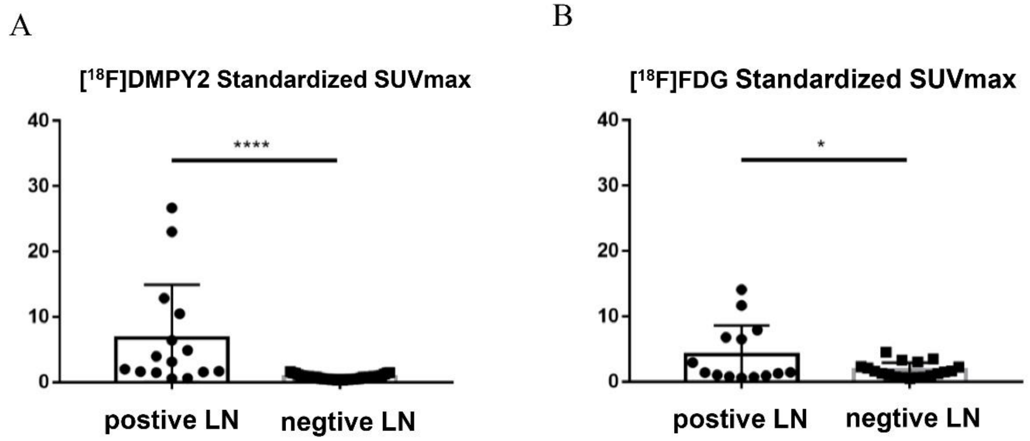

Supplemental Figure 4. The analysis of [^{18}F]DMPY2 Standardized SUVmax (A) and [^{18}F]FDG Standardized SUVmax (B) in metastases LN and non-metastases LN with pathological confirmation.

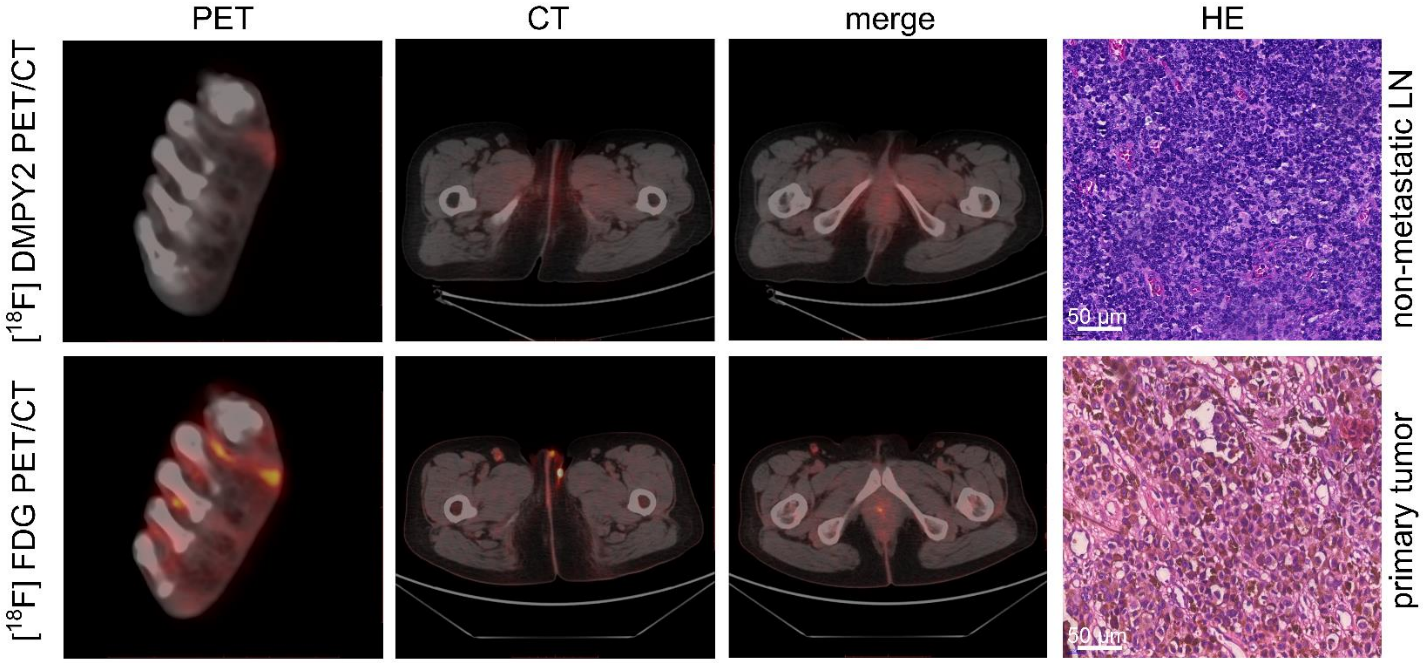

Supplemental Figure 5. A postoperative MM patient (patient #13) with right plantar skin detected by [^{18}F]FDG and [^{18}F]DMPY2 PET/CT. [^{18}F]DMPY2 showed well-defined uptake at the lesion margin, whereas [^{18}F]FDG exhibited confounding uptake due to coexisting tinea pedis. No uptake in the left inguinal lymph nodes on [^{18}F]DMPY2 PET/CT was pathologically confirmed as negative, while [^{18}F]FDG PET/CT showed high uptake. Additionally, hematoxylin and eosin (HE)

staining of both the primary melanoma lesion and the metastatic lymph node further validated the pathological findings.
